# Flexural Strength of Dental Fiber Composite Post Manufactured with a Novel Approach

**DOI:** 10.3390/ma15093370

**Published:** 2022-05-08

**Authors:** Esraa M. Abdelkader, Jose Manuel Cortes Cortes, Candela Reyes Botella, Khaled Nassar, Guillermo Rus, Salma M. Fathy

**Affiliations:** 1 Department of Textile, Faculty of Applied Arts, Badr University, Cairo 11829, Egypt; eng.esraa_mahmoud@outlook.com (E.M.A.); khaled.mansour@gmail.com (K.N.); 2 Department of Structural Mechanics, Ultrasonics Lab (TEP-959), University of Granada, 18071 Granada, Spain; jmcortes@go.ugr.es; 3 Department of Stomatology, Biomedical Group (BIO277), School of Dentistry, University of Granada, 18071 Granada, Spain; creyes@ugr.es; 4 Instituto Investigación Biosanitaria, ibs. Granada, 18012 Granada, Spain; 5 Department of Structural Mechanics, Ultrasonics Lab (TEP-959), Biomechanics Group (TEC-12) and Excellence Research Unit “Modeling Nature” MNat, University of Granada, 18071 Granada, Spain; grus@ugr.es; 6 Department of Dental Biomaterials, Faculty of Oral and Dental Medicine, Zagazig University, Zagazig 44519, Egypt

**Keywords:** fiber composite post, flexural strength, glass fiber, silane coupling, maleic anhydride, thermoplastic resin

## Abstract

Thermoplastic resin fiber composites have an easy fabrication process, good mechanical properties, and compatible stiffness to tooth dentin. However, they have not yet attracted much interest in the field of dentistry. The current study was carried out to test a new proposed approach to manufacture a fiber reinforced composite endodontic post and evaluate its flexural strength through a two-point inclined loading test. The proposed fiber post manufacture approach depends upon a braiding technique of the glass fibers’ (GF) reinforcing component with thermoplastic polypropylene (PP) resin fibers that will later represent the resin matrix after thermal melting. Posts were made of different core (70%) and sheath (30%) construction (PP/GF ratios) using three different GF types and seizing pre-treatment to both fiber types. Two-point inclined loading test at 45 °C applied force angle was used to test the posts’ flexural strength. Fiber posts (1.6 mm in diameter) with pure GF (de-sized starch E-GF and pre-silanized S-GF) core, and sheath construction with higher PP/GF ratios, showed the significantly highest two-point bending strength (56.67 ± 4.89 and 53.96 ± 1.81 MPa, respectively), among experimental posts groups (except for the commercial control posts). However, posts with PP core type showed the lowest values (21.59 ± 1.86 and 16.54 ± 1.94 MPa for de-sized and E-glass sheath fibers, respectively). Based on these findings, the proposed approach was reliable in producing fiber-reinforced composite post with the desired dimensions and fiber distribution. Post construction with a pure GF core and higher PP/GF ratio showed considerably higher flexural strength and GF volume fraction comparable to commercial available post types.

## 1. Introduction

Dental posts are used in the restoration of endodontically treated teeth with a substantial loss of coronary dentin to improve the retention of the final restoration [1]. They can be used to support the core build-up material in such teeth when a ferrule is left over [2]. With the growing aesthetic demand in the field of dentistry, non-metallic posts have gained more popularity over metal ones. They show a lower probability vertical root fracture than metallic posts due to their modulus of elasticity (which is similar to tooth dentin) [3,4]. In addition, they possess good bond strength to radicular dentin, which is also in favor of good stress distribution through the root canals, making them more biomechanically compatible to tooth structure [5,6]. They reported a high flexural strength, reaching 800 MPa [7].

Generally, fiber posts are considered as composite materials composed of three unlike constituents: the resin matrix (continuous phase), the fibers (dispersed phase), and the region in between which is the interphase [8]. These fiber-reinforced composite posts (FRCPs) are manufactured with different fiber types, e.g., carbon, polyaramid, polyethylene, and glass [8], and resin matrices such as epoxy-based, di-methacrylate-based cross-linked matrix, Bis-GMA, or less common aromatic polyimides [9,10]. FRCPs are usually fabricated using pre-stretched reinforcement fibers and pre-treated with silane coupling (organofunctional silanes) agent, which are impregnated into the emulsion of the thermoset polymer as the resin matrix [11,12]. Organofunctional silanes are considered the most effective agent to enhance interfacial adhesion with the resin matrix. Resin-impregnated fibers are heat-cured to form blocks with various forms and diameters. The former blocks are then milled to form the post final shape, a process which may expose some of the fibers onto the surface [10].

Although continuous fiber composite materials with a thermoplastic polymer’s matrix have not been commonly used in dental procedures, they have been demonstrated as cutting edge structural materials. They were reported to show good mechanical properties, excellent and easy processability, low density, recyclability, low cost, and excellent corrosion resistance [13,14]. Various thermoplastic polymers are utilized in the former composite structure such as polypropylene (PP) and polyamide [15,16]. PP is one of the most important industrial petrochemical building blocks. It has a closer coefficient of thermal expansion to glass fibers than thermoset-used resin matrices [10]. Fiber-reinforced PP composites, with glass or carbon fibers, have good durability, moisture resistance, and high strength properties. However, due to the fact that polyolefins such as PP are highly non-polar, they can negatively affect the interfacial adhesion with reinforcing GF. Compounds containing anhydride groups need to be used to increase the surface polarity and interfacial adhesion with the reinforcing phase [17]. The current used approach for fabrication of fiber composite posts has been recently discussed [18]. Braiding of three different types of GF (de-sized stash E-GF, pre-silanized E and S-GF) with thermoplastic PP resins yarns representing the resin matrix were prepared and the FRCPs Young’s modulus was evaluated [18]. The current study focuses on testing the flexural strength of the prepared FRCPs with more technique refinement for the experimental post-manufacturing technique. The experimental hypothesis was that the proposed and refined approach for preparing FRCPs, using different types and percentages of glass fibers and thermoplastic PP resin matrices, will yield a product with comparable flexural properties relative to its commercial peers.

## 2. Materials and Methods

The methodology of the current study was carried out through two stages. The first stage was for testing different percentage combinations of glass fiber/PP (GF/PP) thermoplastic fibers braiding. Materials specification of this stage are mentioned in Table 1. Three main materials categories were used: E-GF treated with starch (ECE225, and count of “22 Tex”) and thermoplastic yarns of polypropylene (PP) with a count of 300 denier, all of which were donated by AGY industries, located in the Aiken, SC, USA. The third category was two types of chemical agents for better coupling and adhesion between two matrices as: Maleic anhydride powder with 99% concentration and tri-methoxysilyl propyl methacrylate with 98% concentration, both purchased from Sigma Aldrich, Steinheim am Albuch, Baden-Württemberg, (Germany), for treating PP yarns and GF surfaces, respectively.

### 2.1. De-Sizing Procedure for Starch Treated E-Glass Fibers (E-GF)

The objective of this treatment was to remove the starch coat form E-GF. It was performed through immersion of the fibers in very low concentration of sulfuric acid (1%) while stirring at boiling temperature (100 °C) for 2–3 min. To ensure that no starch residues were left, a starch iodine test was conducted. First, 0.1 wt% aqueous solution of potassium iodide was prepared by adding 10 gm potassium iodide crystals to 100 mL deionized water then stirring until all crystals dissolve. Then, 5 gm iodine were added with stirring to ensure that no blue color formed which is related to starch presence. All procedures were performed in an opaque container and stored in dark cabinet to avoid light degrading action to the solute.

### 2.2. PP Yarns and E-GF Pre-Treatment (Sizing) before Braiding

The thermoplastic PP yarns were first treated with NaOH (3%) with ratio of 1:100 for 2 h. This was carried out to increase surface area of the yarns so that improve adhesion between the yarns (resin matrix) and glass fibers. Then they were placed in a prepared solution of maleic anhydride (1 g/100 mL) and heated 120 °C in an oven (Hobersal, Barcelona, Spain) for two hours. The maleic anhydride/filaments mass proportion was 10% [19,20]. For GF surface treatment, 1 wt% aqueous solution of silane coupling agent, with pH adjusted to 4 using acetic acid, was prepared. GF fibers were dipped within the silane solution then squeezed with squeezing rolls. Afterwards, they were dried in the previously mentioned oven for 10 min at 110 °C [21].

### 2.3. Fibers Braiding and Post Fabrication

Fiber-reinforced composite posts (FRCPs) with dimensions of 1.6 mm diameter and 2 cm length were fabricated. They consisted from a core (70% of the post’s fibers volume fraction) and sheath (30% of the post’s fibers volume fraction). There were three main groups according to the post core type; pure PP, pure GF and mixed GF/PP (50/50%) cores. Each group was divided into six subgroups according to the sheath braiding construction (fiber volume fraction within post sheath) of PP/GF fibers as: 90/10%, 80/20%, 70/30%, 60/40%, and 50/50%, respectively. Additional two groups of commercial FRCPs were used as control groups; Olipost Light with 1.6 mm diameter (Olident, Cologne, Germany) and SF radiopaque post 1.6 mm in diameter (IndiaMart, Intermesh LTD, India). The different braiding constructions to form different cores and sheaths for the post were performed using the two gears of 42 teeth’s tension gear and 20 teeth’s draw gear meshing. The former gears allow for higher torque transmission to the braiding yarns consequently obtaining the tightest structure of the braid with the most acute and closed braiding angle (45°), and the least radius for the produced braids. The number of working spindles for braiding was 14 ± 1 for creating the suggested ratios of thermoplastic ones (PP) and the reinforcement yarns (GF). Additionally, an extra-sheath of PP 16 thermoplastic yarns was used to cover the whole formed posts external surface.

Afterwards, the produced braids were cut and positioned in split aluminum mold containing grooves, each groove would be of 1.6 mm tube diameter when the two mold parts close over each other. The mold containing the post braids was then placed into a digital oven (Hobersal, Barcelona, Spain) for complete melting of thermoplastic PP yarns at 165 ± 5 °C for 40 ± 5 min. After mold complete cooling, the produced posts were removed from the grooves using a thin needle and then they were ready for mechanical testing.

The second stage was carried out after mechanical testing of the posts produced from the first stage using the braid construction with best mechanical results. Two additional types of GF were used to construct FCRPs using three types of GF through this stage. They were; the former G-GF de-sized starch type, E-GF (ECDE75) with a count of “66 Tex” and S-GF (SCG75) with a count of “68 Tex”. The latter two types were pre-silanized and donated by AGY industries, located in the Aiken, SC, USA.

**Figure 1:**
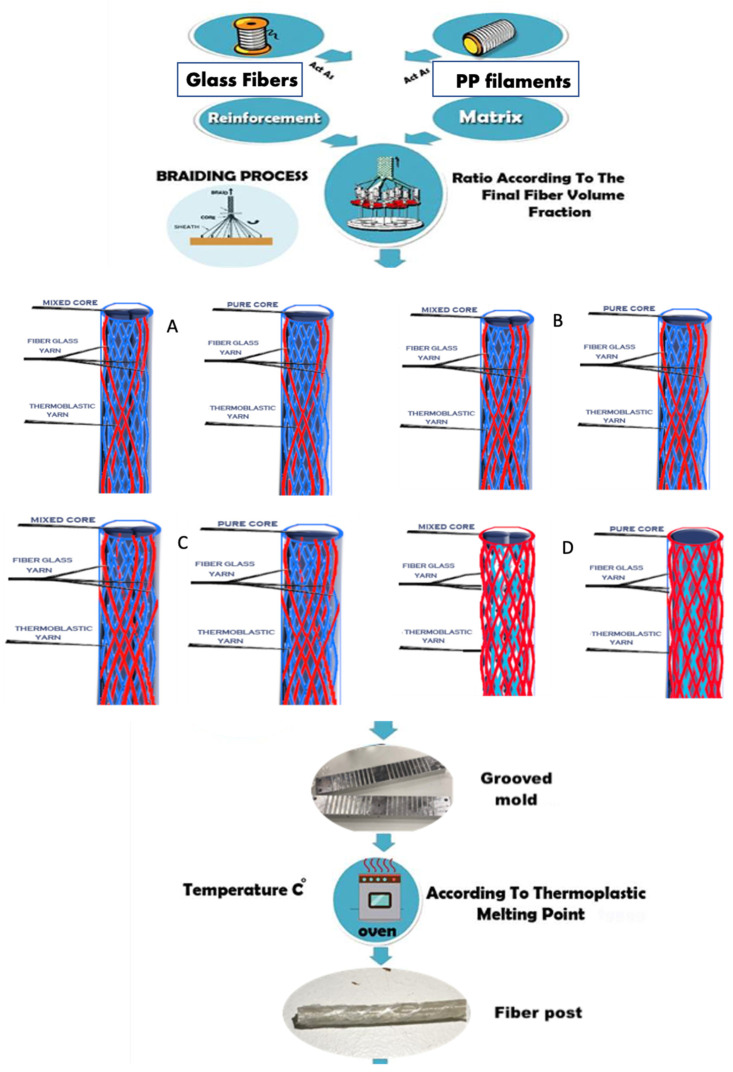
Schematic representation for the steps of experimental post production, (**A**–**D**) represent different core construction (mixed and pure) and the different sheath composition with increase in GF% (red lines) in relation to PP fibers % (blue lines) from (**A**–**D**).

The post construction procedures, within this stage, was the same as previously described in the first one, using the three core constructions and the sheath braid with PP/GF percentage of 90/10. The former percentage produced the best mechanical results within the first stage. Figure 1 illustrates the steps for experimental post preparation.

### 2.4. Two-Point Inclined Loading Test (Compression at 45°)

**Figure 2:**
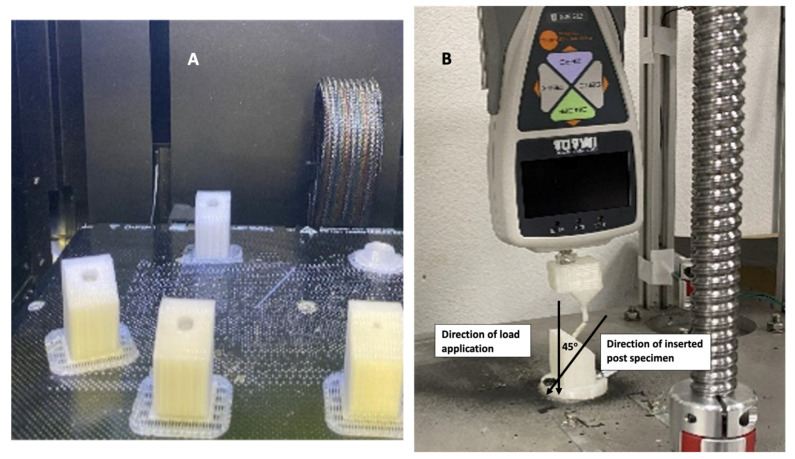
Two-point bending strength (**A**) 3D printed specimen holder, (**B**) The applied force on the fiber post in a way to form a vector force of 45° as two-point bending stress.

540 experimental fiber-reinforced composite posts (FRCPs), for the first stage testing, and 90 posts, for the second stage, in addition to two groups of commercial FRCPs (n = 10). Specimens’ grips were fabricated using 3-dimentional (3D) printer (UP BOX, Beijing Tiertime Technology Co., Ltd., Beijing, China). Poly-lactic acid polymer filament (1.75 mm, White, ANYCUBIC, China) was used to create the 3D printed holders (Figure 2A). The specimens were fixed in a pre-fabricated hole within the 3D model, which has the same diameter as the tested fiber post, perpendicular to the inclined top-surface of the holder (Figure 2B). The load was applied on the post top surface in a way that the vectorial force forms 45° angle with the post long axis (Figure 2B).

The test was conducted using a universal testing machine (Instarus universal testing machine, Spain) using a load cell of 500 N at cross-head speed of 1 mm/min to the incisal surface of the post until fracture of the specimen.

The stress (τ in MPa) at fracture was obtained through the following formula [22]:

τ = 16. Fmax. cos 45/ 3πD ^Ç^ (1)

where Fmax is the maximum force at fracture (N); cos 45, refers to a cosine angle of 45°; π is 3.14; and D the diameter of the post at the fulcrum or deflection point (1.6 mm for experimental post). The broken post surfaces were then coated with conductive carbon and examined using SEM (ZEISS Gemini SEM 560, Oberkochen, Germany).

### 2.5. Statistical Analysis

The data were first evaluated for normality through “Shaprio–Wilk” statistical test. The data were tabulated for statistical analysis using statistical, package SAS 9.1.3. Means and standard deviations were calculated and expressed in MPa. Data were statistically analyzed using two and one-ANOVA followed by *Tukey’s* post hoc test (α = 0.05).

## 3. Results

**Figure 3:**
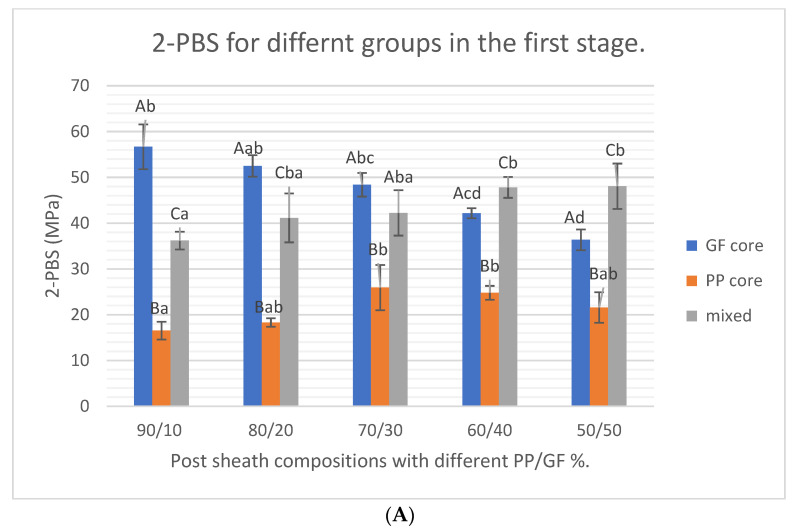

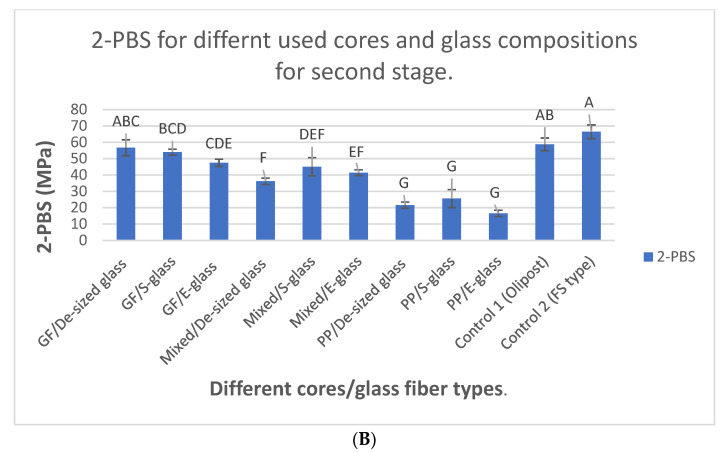
Bar charts showing the 45° angle compression testing (two-point bending strength) for first stage (**A**) with highest values were for GF cores in higher PP/GF % core composition and (**B**) for the second stage where de-sized Gf and S- glass fiber cores showed the closest two-point bending strength values to control 1 and 2 commercial post types. Upper and/or lowercase letters are for *Tukey’s* post-hoc test.

The results of the current study showed that within the first stage both the type of post core and the interaction between both core type and the sheath composition % had a statistically significant effect on the two-point flexural strength (*p*-value < 0.0001). On the other hand, the sheath composition % did not have statistically significant effect (*p*-value = 0.2147) (Table 2 and Figure 3).

The results of the first stage through this study showed statistically significant (*p*-value < 0.0001) highest two-point bending flexural strength for posts with GF cores and core sheath structure of 90/10 and 80/20 % (PP/GF) as 56.67 ± 4.89 and 52.49 ± 2.36 MPa, respectively. Fiber posts with mixed core type showed statistically higher two-point bending strength (*p*-value = 0.0280) with increasing the GF % within the sheath (60/40 and 50/50 % of PP/GF). Posts with PP core type showed the statistically significant lowest flexural strength values within all core construction % (Table 3 and Figure 3). Within the second stage, again fiber composite posts with GF core showed the statistically significant highest two-point bending flexural strength values (*p*-value < 0.0001) with the highest values for de-sized starch E-GF and pre-silanized S-GF (56.67 ± 4.89 and 53.96 ± 1.81 MPa, respectively) followed by and pre-silanized E-GF (47.48 ± 2.2 MPa). However, they all showed statistically significant (*p*-value < 0.0001) lower flexural strength values than two commercial types (66.44 ± 4.27 and 58.73 ± 3.90 for control 2 and control 1, respectively) (Table 4).

**Figure 4:**
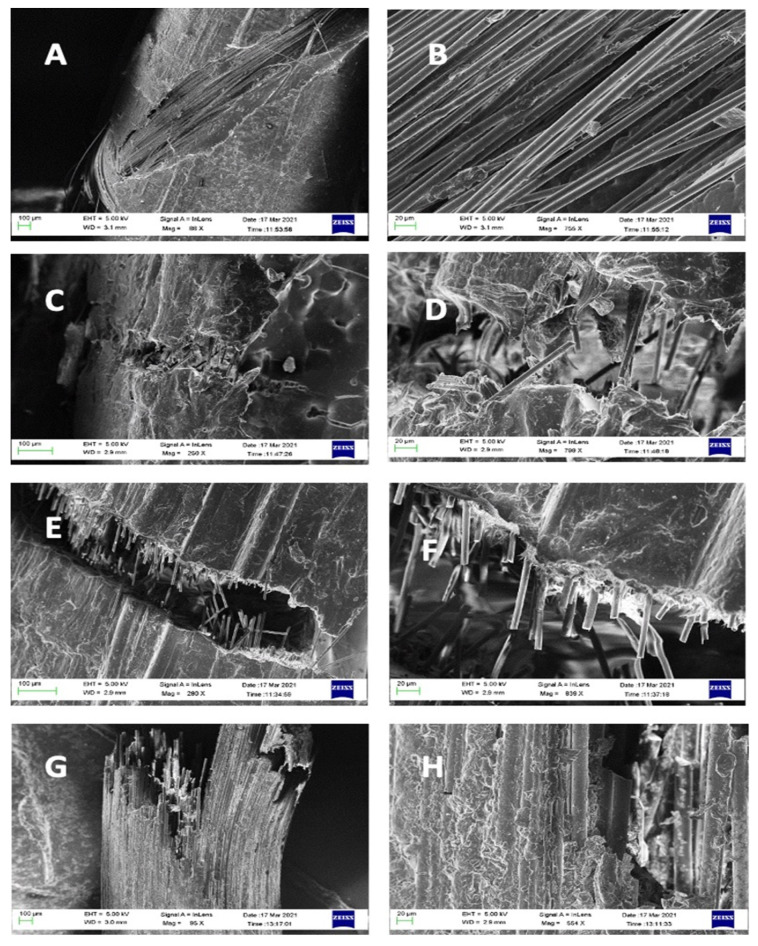
SEM images of FRCPs after two-point bending test showing, (**A**,**B**) fiber post with pure GF core and sheath composition of 80/20% (PP/GF) with cracking on the surface and most of fibers appear intact and parallel to long axis of the fiber post (**C**,**D**) fiber post with mixed core and S-GF glass fiber showing once breakage within the sheath goes easier within core due to less GF content, (**E**,**F**) showing fiber post with pure PP core and sheath with 80/20% (PP/GF). They show almost behavior such as the later type with mixed core, (**G**,**H**) are the commercial control type 1 (Olipost) the core showing almost breakage of all-GF after reaching the maximum high bending stress.

The SEM images showed cracking within resin matrix on the surface and cutting within GF that is caused under tensile stress during bending stress. They show slight deformation of the GF especially within control 1 (Olipost) which could happened prior to failure under tensile stresses within bending (Figure 4G,H). Other images within Figure 4 (e.g., C–F) showed apparent less volume fraction of GF within the resin matrix which may cause decrease within flexural strength even though they appear parallel to the long axis of the fiber post.

## 4. Discussion

The current study evaluated the flexural strength to fiber-reinforced composite posts (FRCPs) fabricated with new approach. The former approach depends on the braiding of GF with the thermoplastic fibers of the resin matrix which may allow for better controlling of the GF distribution within the matrix. PP thermoplastic resin was used as the thermoplastic resin matrix. It is known for its easy processability and low coefficient of thermal expansion (0.6–1.7 × 10^−6^/°C) which is closer to E-glass fibers coefficient (8 × 10^−6^/°C) than the reported thermoset resin matrices (40–80 × 10^−6^/°C) [10]. That may improve the long-term integrity of the constructed fiber post.

The results of the current study showed significantly highest flexural strength values for FRCPs with GF cores and sheath with higher PP/GF ratio in both first and second stages of the research. The lowest flexural strength values were for FRCPs with a PP core. This may be attributed to the highest GF volume fraction in experimental FRCPs with GF core (around 70% of the fiber volume within fiber core) while the total GF volume fraction could reach around 74% of the whole core and sheath in posts with higher PP/GF ratio of the sheath. This former explanation goes hand in hand with what was reported previously, as increasing the fiber density or fiber volume fraction was a contributing factor to increasing mechanical properties such as elastic modulus and yield stress [23]. The current suggested post configuration allowed for loading of such high percentage of GF in the form of pure core made of GF that was then held by the sheath made of both GF and melted PP matrix. The diameter of used GF, within this study, were of 6–9 microns, which are within the range reported previously as 6–21 microns for GF in fiber posts [7,24]. Decreases within the GF diameter allows for higher packing density of the used fibers (up to 70%) [10].

Although the tested commercial types, control 1 and 2, showed the significantly highest flexural strength values, the highest results introduced by experimental FRCPs are comparable to previous study [22]. The later study reported using FRCPs type of up to 80% GF volume fraction within the post. However, the control 1 type (Olipost), as reported by the manufacturer, was composed of 68% GF, 19% nano-zirconia particles and the rest of a 32% resin matrix) [25]. The filler nano-loading may cause further enhancement with the commercial post mechanical properties. That could be considered as limitation with the current proposed FRCPs manufacture approach. It is recommended the one further modify the proposed approach to allow for various filler particles incorporation for higher mechanical performance of FRCPs in future studies.

Both the core composition and of the interaction between both the core the sheath composition showed significant impact on the fabricated posts flexural strength. However, the sheath composition alone did not have a significant impact on the post strength. That may be correlated to the proposed structure of the post with 70% volume related to the core part. The groups with higher thermoplastic content, such as in the case of pure PP core and mixed core with higher PP/GF ratio in the sheath, showed the lowest flexural strength. These results are in agreement with recent study which concluded that higher thermoplastic content of PP in the fiber- reinforced composites had more inhomogeneous distribution of reinforcement fibers [26]. This high content caused unexpected rise to viscoelastic properties of the composite with decrease in mechanical properties [26].

Similarly, when different GF types used, within the second stage, posts with a GF core still showed the highest flexural strength values. FRCPs with starch seized E-glass fibers that received surface pretreatment to PP yarns and GF showed almost higher flexural strength than pre-silanized S-glass and E-glass fibers. One of the factors that may improve the adhesion between PP resin matrix and silanized GF was silanization of GF, in aqueous solution, with silane coupling with general structure [X-Si(OR)_3_]. The R represents an alkyl group and where X group is still available to react with reactive functions of the polymer which is known for interaction with thermoset resins [27,28]. However, as this approach suggests using thermoplastic resin as fiber resin matrix, surface seizing with maleic anhydride to increase polarity of thermoplastic PP (polyolefins) was carried out. The previous treatment was reported to improve adhesive bond with the amine group of the silanized glass surface [29]. From a microscopic point of view, the reinforcing fibers prevent crack propagation by chemically bonding to the polymer matrix with covalent bonds [30]. Nevertheless, posts with seized GF and pre-silanized S-GF showed comparable results to previous two-point bending flexural results [22]. This may be attributed to the higher tensile strength and modulus with higher silica content (up to 64 and 24 wt% silica and alumina, respectively) of S-GF than commercially available E-GF [31]. The limitations of the current study were, first, the absence of introducing filler particles within the resin matrix. Second, using 3D printed polymer to act the substructure surrounding the post during mechanical testing instead of using natural tooth which is not the closer situation to the oral cavity. Therefore, it is suggested that future researchers further improve the technique of the suggested fiber post manufacture. Testing other thermoplastic resin matrices as well as incorporating reinforcing filler particles with nanosized particles are also suggested.

## 5. Conclusions

Based on the results and within the limitations of the current study the following conclusions can be inferred. The proposed approach was successful for fiber post manufacture with the desired dimensions and structure. The fiber posts with a higher GF volume, e.g., pure GF core and higher PP/GF % ratio showed the significantly highest flexural or two-point bending strength values (except for the used commercial types). The de-sized starch E-glass fiber and pre-silanized S-GF showed, with the GF core, the best results for flexural strength for tested posts.

## Figures and Tables

**Table 1:** Glass fibers and polypropylene yarns specifications.

Product	ECE225 (Starch Treated)	SCG75 (Pre-Silanized)	ECDE75 (Pre-Silanized)
Glass Type	E	S–2 GLASS	E
Filament Diameter (microns)	7	9	6
Nominal Yield—yd/lb.	22,500	7295	7500
Tex—g/1000 m	22	68	66.1
Tex tolerance +/−	1.2	6.2	4.3
Nominal Solids %	1.4	1.17	1.42
Solids Tolerance +/−	0.25	0.26	0.17
Nominal Twist TPI (TPM)	0.5Z (Z20)	1.0Z (Z40)	0.7Z (Z28)
Twist Tolerance +/− TPI (TPM)	0.15 (6)	0.3 (12)	0.21 (8)
Max. Broken Filaments	10	9	10
Approximate Yarn Diameter—in (mm)	0.0065 (0.165)	0.0076 (0.192)	0.106 (0.269)
Yarns Type		Polypropylene (PP)	
Count		300 Denier	
Melting point		165 °C	
Young’s Modulus (GPa)		1.38	
Tensile strength (MPa)		34	

TPI, turns per inch; TPM, turns per meter; and yd/lb, yards/pounds.

**Table 2:** Two-way ANOVA showing the effect of both post core type and sheath composition percentage on two-point bending flexural strength (Compression at 45°) of fiber composite post.

Source of Variance	DF	Sum of Squares	Mean Squares	F-Value	*p*-Value
Post core type	2	5760.796084	2880.398042	251.51	<0.0001 *
Sheath composition %	4	70.743676	17.685919	1.54	0.2147
Post core type X Sheath composition %	8	1207.832871	150.979109	13.18	<0.0001 *
Error	30	343.576867	11.452562		
Total	44	7382.949498			

* means statistically significant at *p*-value ≤ 0.05.

**Table 3:** Means and standard deviations (SD) of two-point bending flexural strength (2-PBS) (MPa) for first stage.

Post Sheath Composition (PP/GF%)	2-PBS			*p*-Value
	GF Core Type	PP Core Type	Mixed (50/50%) Core Type	
	Mean ± SD	Mean ± SD	Mean ± SD	
90/10	56.67 ± 4.89 ^Aa^	16.54 ± 1.94 ^Ba^	36.18 ± 1.95 ^Ca^	<0.0001 *
80/20	52.49 ± 2.36 ^Aab^	18.29 ± 0.93 ^Bab^	41.14 ± 5.34 ^Cba^	
70/30	48.39 ± 2.59 ^Abc^	25.91 ± 4.95 ^Bb^	42.24 ± 4.96 ^Aba^	0.0018 *
60/40	42.17 ± 1.11 ^Acd^	24.78 ± 1.52 ^Bb^	47.81 ± 2.28 ^Cb^	<0.0001 *
50/50	36.36 ± 2.28 ^Ad^	21.57 ± 3.34 ^Bab^	48.06 ± 4.95 ^Cb^	0.0004 *
*p*-value	<0.0001 *	0.0116 *	0.0280 *	

Letters are for *Tukey’s test*, a–d = Means with same small letter in each column are not significantly different, A–C = Means with same capital letter in each row are not significantly different, * means there is significant difference at *p*-value ≤ 0.05.

**Table 4:** Means and standard deviations (SD) of two-point bending flexural strength (2-PBS) (MPa) for second stage.

Group Core Type/GF Type	2-PBS
	Mean ± SD
GF/De-sized glass	56.67 ± 4.89 ^ABC^
GF/S-glass	53.96 ± 1.81 ^BCD^
GF/E-glass	47.48 ± 2.22 ^CDE^
Mixed/De-sized glass	36.18 ± 1.95 ^F^
Mixed/S-glass	45.05 ± 5.55 ^DEF^
Mixed/E-glass	41.36 ± 1.77 ^EF^
PP/De-sized glass	21.59 ± 1.86 ^G^
PP/S-glass	25.54 ± 5.55 ^G^
PP/E-glass	16.54 ± 1.94 ^G^
Control 1 (Olipost)	58.73 ± 3.90 ^AB^
Control 2 (FS type)	66.44 ± 4.27 ^A^
*p*-value	<0.0001

Letters are for *Tukey’s test*, A–G = Means with same letter in are not significantly different.

## Data Availability

Not applicable.
